# Ice-Binding Proteins in Plants

**DOI:** 10.3389/fpls.2017.02153

**Published:** 2017-12-22

**Authors:** Melissa Bredow, Virginia K. Walker

**Affiliations:** ^1^Department of Biology, Queen’s University, Kingston, ON, Canada; ^2^Department of Biomedical and Molecular Sciences, and School of Environmental Studies, Queen’s University, Kingston, ON, Canada

**Keywords:** ice-binding protein, antifreeze protein, plant freezing stress, freeze tolerance, ice-recrystallization inhibition

## Abstract

Sub-zero temperatures put plants at risk of damage associated with the formation of ice crystals in the apoplast. Some freeze-tolerant plants mitigate this risk by expressing ice-binding proteins (IBPs), that adsorb to ice crystals and modify their growth. IBPs are found across several biological kingdoms, with their ice-binding activity and function uniquely suited to the lifestyle they have evolved to protect, be it in fishes, insects or plants. While IBPs from freeze-avoidant species significantly depress the freezing point, plant IBPs typically have a reduced ability to lower the freezing temperature. Nevertheless, they have a superior ability to inhibit the recrystallization of formed ice. This latter activity prevents ice crystals from growing larger at temperatures close to melting. Attempts to engineer frost-hardy plants by the controlled transfer of IBPs from freeze-avoiding fish and insects have been largely unsuccessful. In contrast, the expression of recombinant IBP sequences from freeze-tolerant plants significantly reduced electrolyte leakage and enhanced freezing survival in freeze-sensitive plants. These promising results have spurred additional investigations into plant IBP localization and post-translational modifications, as well as a re-evaluation of IBPs as part of the anti-stress and anti-pathogen axis of freeze-tolerant plants. Here we present an overview of plant freezing stress and adaptation mechanisms and discuss the potential utility of IBPs for the generation of freeze-tolerant crops.

## Introduction

Plant growth is highly dependent on temperature, which dictates both the geographical range in which plants can be grown and potential yield. Apart from tropical zones, sub-zero temperatures can occur almost everywhere, making freezing damage a global concern. Plants vary in their “cold-hardiness” depending on the level of low temperature stress they can tolerate. Many temperate agricultural crops, including tobacco, tomatoes, potatoes, corn and apples, as well as certain ornamentals such as impatiens, begonias and phlox are considered “very tender” or “half-hardy” and sustain freezing damage between 0 and -4°C. As such, sub-zero temperatures in early spring and late autumn not only shorten the growing season but also result in substantial crop loss. For example, approximately 2 billion USD were lost in citrus crops due to a single frost event in California in 2013 (United States Department of Agriculture, National Agricultural Statistics Service). More recently, a spring frost in Austria resulted in a devastating 80% loss in the total fruit harvest resulting in more than 100 million Euro in damages (Statistics Austria).

**FIGURE 1:**
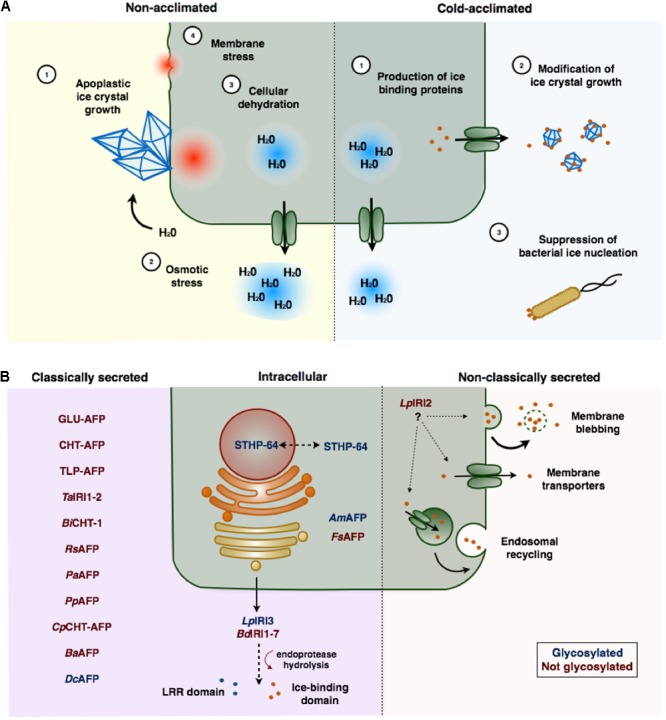
**(A)** Freezing stress and ice-binding proteins (IBP)-induced freeze protection in plants. In the absence of IBPs, large ice crystals form in the apoplast that can physically damage plasma membranes (1). As water molecules join the ice crystal lattice an osmotic gradient is formed (2) resulting in the sequestration of intracellular water, and consequent cellular dehydration (3). The loss of cell volume may cause cells to collapse or rupture (4). Cold acclimation induced expression of IBPs, which are typically secreted to the apoplast (1), adsorb to seed ice crystals preventing their growth (2). IBPs may also prevent freezing associated with bacterial ice nucleation (3). **(B)** Localization and post-translational modification of ice-binding proteins in plants. Most plant IBPs contain amino-terminal secretion signals and are localized to the apoplast through the endoplasmic reticulum (ER)–Golgi apparatus pathway. Some of these proteins (LpIRI3 and BdIRI1-7), appear to be hydrolyzed following secretion, releasing an LRR domain from the ice-binding domain. LpIRI2, which lacks a signal peptide, is secreted, likely through a non-classical secretion pathway. Few IBPs have been localized to the intracellular space. Glycosylated proteins are indicated in red, proteins that are not glycosylated are indicated in blue. IBPs are named according to **Table 1**.

Damage to plant cells invariably results following the growth of ice crystals in the extracellular space (**Figure 1A**; Guy, 1990; Pearce and Ashworth, 1992; Wisniewski et al., 1997). The growth of large ice crystals, at the expense of smaller ones, occurs through a thermodynamically favorable process known as ice recrystallization. Cells may sustain mechanical damage or cellular dehydration resulting from the sequestration of intracellular water caused by the osmotic imbalance imposed by the exclusion of solutes from recrystallized ice (Steponkus, 1984; Pearce, 2001). This can have devastating effects on cell metabolism associated with protein inactivation or denaturation and an increase in reactive oxygen species (Thomashow, 1998). If the dehydration is severe, the loss of cell volume may also facilitate the collapse of membrane structures resulting in cell lysis.

To prevent frost damage, growers can use passive approaches including optimal site selection, land clearing, soil management or choose crop varieties that are less temperature sensitive. Alternatively, they may employ active approaches such as covering crops, use of smoke clouds, wind machines, water sprinkling or heating (Lindow, 1983). However, these methods have their own limitations and require quick execution. In order to decrease the probability of freezing, growers may instead choose to sow crops later in the season, though delaying planting has its own risks including a decrease in potential yield. Therefore, it is important that we develop new technologies to prevent freezing damage, such as exploring protection mechanisms adapted by freeze-tolerant plants, which have potential to contribute to the development of more hardy crops.

Ice-binding proteins (IBPs) are one such family of low temperature-associated proteins found in certain cold-hardy animals and microbes as well as some freeze-tolerant plants. These proteins serve to control the growth of ice crystals and mitigate freezing damage (**Figure 1A**). Plant IBPs appear to be particularly efficient at adsorbing to ice crystals, thereby preventing the migration of water molecules at temperatures close to freezing or under freeze-thaw conditions. This property is known as ice recrystallization inhibition (IRI). Because of these functional properties, plant IBPs have also been variously known as antifreeze proteins (AFPs) or IRI proteins.

Plant IBPs have likely evolved independently many times (Gupta and Deswal, 2014b). This diversity has resulted in a multiplicity of other functions associated with biotic and abiotic stress responses, including most relevantly, the inhibition of bacterial ice nucleation which would otherwise result in freezing at high sub-zero temperatures (Tomalty and Walker, 2014; Bredow et al., 2017). Indeed, the wide-distribution of IBPs across a variety of plant species points to their importance in freezing tolerance and also highlights their possible utility for the development of freeze-tolerant crops.

## Low Temperature Stress and Adaptation

### Ice Nucleation and Freezing Stress in Plants

The site of ice nucleation, as well as the temperature at which nucleation occurs, is a major determinant of the measure of freezing injury. Ice crystals form when water molecules join to produce an “ice-nucleus”, serving as an initiation point for surrounding water to form an ice crystal lattice. Homogeneous nucleation can occur spontaneously at very low sub-zero temperatures (below -40°C). However, ice crystal formation occurs at higher temperatures by heterogeneous nucleation, catalyzed by the presence of dust, salts, organic molecules or ice-nucleation active (INA) bacteria (Pearce, 2001). Apart from ice itself, INA bacteria are the most efficient heterogeneous nucleators known. Plants are not completely vulnerable, however, and have the capacity to supercool their fluids, in some cases to temperatures lower than -5 or -10°C (Arny et al., 1976; Marcellos and Single, 1979; Lindow, 1983). Nevertheless, nucleation can occur intrinsically, or extrinsically on the surface of plant tissues, with ice gaining entry through stomatal openings, hydathodes, or through cracks in the surface of the cuticle (Ashworth and Kieft, 1995; Wisniewski and Fuller, 1999; Pearce and Fuller, 2001).

In plants, liquids are generally contained in two compartments: the symplast, which encompasses water in the cytosol and central vacuole, and the apoplast consisting of water in the xylem-lumen space, the cell wall and the extracellular space surrounding cells (Canny, 1995). Since the apoplast generally has a higher freezing temperature, and is the first point of access for extrinsic ice nucleators, ice crystals typically appear first in the apoplastic space. However, if temperatures drop rapidly, intracellular ice crystals may also form, causing irreversible cell damage (Steponkus, 1984). Thus, the apoplast acts a boundary between vital intracellular components and the external environment, and ice nucleation must be limited to the apoplast for survival.

### Cold Acclimation

Perennials, biannuals, and hardy annuals from temperate and polar regions have evolved means to adapt to low temperatures without sustaining significant damage in order to overwinter, produce seeds and/or survive seasonal frosts. Biochemical, metabolic, and physiological changes that allow plants to withstand low temperature stress are acquired through exposure to low temperatures and/or shortened day length prior to freeze events, a process known as cold acclimation (Thomashow, 1999). Typically, such changes are induced in the autumn to prepare for the approaching winter season.

The degree to which a plant can acclimate is based largely on the underlying genetic background, as well as environmental conditions, including the minimum temperature and duration of cold exposure. Perhaps not surprisingly, harsher environmental conditions can result in a greater capacity to acclimate to low temperatures. For example, following cold acclimation, the generally frost-sensitive mustard *Arabidopsis thaliana*, has a maximal freezing tolerance of approximately -12°C, compared to -5°C observed in non-acclimated plants (Wanner and Junttila, 1999). In contrast, cold-acclimated frost-hardy winter wheat and forage grasses survive below -25°C, while some plants from Arctic and alpine regions can withstand temperatures below -50°C (Sakai and Larcher, 1987; Moriyama et al., 1995; Yoshida et al., 1997). It should be noted that temperature is not the only variable affecting cold acclimation; freeze-tolerance capabilities are altered by both photoperiod (Fuchigami et al., 1971; Wanner and Junttila, 1999; Welling et al., 2002) and the developmental stage at the time of exposure (Mahfoozi et al., 2001).

The level of freeze protection is largely determined by a plant’s ability to protect plasma membranes from injury. For this reason, modifications to the plasma membrane are amongst the first changes observed after cold acclimation. These include alterations in the composition of lipid constituents towards a larger proportion of unsaturated fatty acids (Steponkus et al., 1988) as well as changes in protein composition, which alter signal transduction pathways and allow membranes to interact with cryoprotective proteins (Uemura and Yoshida, 1984). As cellular dehydration is one of the most damaging consequences of freezing, metabolism is redirected towards the production of low molecular weight cryoprotectants including fructan, proline, and glycine betaine, as well as soluble sugars (namely raffinose and trehalose) and sugar alcohols (sorbitol and inositol) (Hughes and Dunn, 1996; McNeil et al., 1999; Thomashow, 1999; Hincha et al., 2000; Krasensky and Jonak, 2012; Abeynayake et al., 2015). The accumulation of millimolar quantities of these compatible solutes in the cytoplasm helps regulate the osmotic balance between cellular compartments in order to combat cellular dehydration.

Changes in gene expression are critical to regulating and maintaining cold acclimation. As an early response to low temperature, DNA methylation occurs with hypermethlylation providing stability to DNA (Kovalchuk et al., 2003). However, throughout cold acclimation, dynamic changes in DNA methylation are observed (Baulcombe and Dean, 2014) associated with genome wide changes in expression including the upregulation of such protective proteins as cold-regulated proteins (Thomashow, 1998), cold-shock domain proteins (Sasaki and Imai, 2012), dehydrins and other late embryogenesis-associated proteins (Thalhammer and Hincha, 2013; Thalhammer et al., 2014; Nakayama et al., 2014; Takahashi et al., 2014), and heat shock proteins (Zhang et al., 2008). Other changes, including epigenetic modifications, are critical for the stabilization of membranes, scavenging of reactive oxygen species and maintaining enzyme activity. IBPs, which are also induced by cold acclimation, limit cellular damage associated with freezing and have been reported, although not fully characterized, in dozens of plants. Upregulation of plant defense mechanisms and pathogenesis-related (PR) proteins (Gaudet et al., 2003) are also important, given the increased risk of infection by psychrotolerant microbial species, which themselves may have freeze-protective mechanisms.

### Ice-Binding Proteins and Freeze Protection

Ice-binding proteins are functionally and structurally distinct and have been identified across a number of biological kingdoms with species as diverse as fish (DeVries et al., 1970), insects (Duman and Patterson, 1978; Duman, 2015), algae (Raymond et al., 2009), microbes (Gilbert et al., 2004), and plants (Griffith et al., 1992). An invariable feature of these proteins is their unique ability to adsorb to ice and modify its growth. Having evolved independently multiple times, it is not surprising that IBPs have high functional and structural diversity (Davies, 2014; Bar-Dolev et al., 2016). In some cases, IBPs have secondary functions not obviously associated with freezing point depression including PR activities (Griffith and Yaish, 2004), transcriptional regulation (Huang and Duman, 2002), membrane stabilization (Tomczak et al., 2002), ice adhesion (Guo et al., 2012), and ice structuring (Raymond, 2011). The ice binding properties of an IBP can be, at least partially, explained by the survival strategy employed by the host. Some organisms have adopted a freeze-avoidance approach and allow their fluids to supercool below the temperatures normally encountered in their environment, thereby avoiding ice crystal formation. In contrast, a freeze-tolerance survival strategy permits some ice crystal growth while inducing mechanisms to prevent the damage associated with freezing stress. Plants may adapt either of these strategies. For example, seeds may avoid freezing by overwintering with little water content or by depressing the freezing point through the accumulation of sugars and polyols (Wisniewski et al., 2014). Alternatively, plants can tolerate freezing by cold acclimating and using IBPs to protect cells (Thomashow, 1998).

Ice-binding activity was identified more than 50 years ago, with the initial observation of freezing point depression in an insect (Ramsay, 1964), followed by the characterization of an antifreeze glycoprotein in Antarctic fish (DeVries et al., 1970). IBPs have traditionally been categorized based on their ability to suppress the freezing point of solutions in relation to the equilibrium melting point, generating a thermal hysteresis (TH) (DeVries et al., 1970). Most IBPs identified in fish are moderately active, with TH activities up to 2°C, sufficient to prevent the lethal freezing of interstitial fluids in ice-laden oceans (Fletcher et al., 2001). Hyperactive IBPs identified in some insects, terrestrial arthropods, bacteria, and some polar fish, are able to suppress freezing points by 2–13°C (Scotter et al., 2006). Thus, IBPs from these groups of organisms are typically known as AFPs. In contrast to these examples, plant IBPs have low TH, typically in fractions of a degree.

Plant IBPs were discovered 25 years ago with their description in winter rye (Griffith et al., 1992). As indicated, plant proteins have low TH activities (0.1–0.5°C, Griffith and Yaish, 2004; Gupta and Deswal, 2014b). A notable exception are those reported, but not yet characterized, from spruce (*Piceae* sp.) with TH activities of ∼2°C (Jarzabek et al., 2009). Since supercooling can promote intracellular ice formation and rapid, potentially catastrophic ice crystal growth, freezing at temperatures closer to the equilibrium freezing point results in higher survival (Gusta and Fowler, 1977; Gusta et al., 2004). Thus, it is reasonable that plants appear to favor IBPs with low TH that may allow more controlled ice crystal growth (Levitt, 1980). Despite their characteristic low TH, many plant IBPs are notable for their superior ability to restrict ice crystal growth at micromolar concentrations (as low as 3 μg/mL for *Lp*AFP; Lauersen et al., 2011) (**Table 1**), a property known as IRI (Knight and Duman, 1986). A non-proteinaceous glycolipid has also been identified in bittersweet nightshade, *Solanum dulcamara*, in addition to a glycosylated IBP. The glycolipid, which is composed of repeats of mannose-xylose disaccharides, has relatively high TH activity (∼ 3°C; Walters et al., 2011).

**Table 1:** Activity of identified ice-binding proteins in plants and their similarity with other proteins.

Protein	Plant of origin	Ice-binding activity	Similarity	Reference
GLU-AFP, CHT-AFP, TLP-AFP	Winter rye (*Secale cereale*)	TH = 0.03°C at 0.1 mg/mL, hexagonal bipyramidal crystals	Two β-1,3-endoglucanases, one class-I endochitinase, one class-II endochitinase, two thaumatin-like	Hon et al. (1994, 1995);Yeh et al., 2000
STHP-64	Bittersweet nightshade (*Solanum dulcamara*)	TH = ∼0.3°C at >30 mg/mL, high levels of IRI	WRKY transcription factor	Duman (1994);Huang and Duman (2002)
*Dc*AFP	Carrot (*Daucus carota*)	TH = 0.35°C at >1 mg/mL, high levels of IRI	Poly-galacturonase inhibition protein	Worrall et al. (1998);Smallwood et al. (1999)
*Lp*AFP, *Lp*IRI2/3	Perennial ryegrass (*Lolium perenne*)	TH = ∼0.3°C at 1.5 mg/mL, high levels of IRI	Phytosulfokine receptor kinase	Sidebottom et al. (2000); Bredow et al. (2016a)
*Am*AFGP	Mongol Menkhargana (*Ammopiptanthus mongolicus*)	TH = 0.15°C at 5 mg/mL and 0.35°C at 10 mg/mL, hexagonal bipyramidal ice shaping, IRI activity	Agglutinin	Fei et al. (2001); Wang et al. (2003);Fei et al. (2008)
*Fs*AFP	Weeping forsythia (*Forsythia suspensa*)	IRI activity at very low concentrations (6 μg/mL)	Dehydrin-like protein	Simpson et al. (2005)
*Ta*IRI1-2	Winter wheat (*Triticum aestivum*)	High IRI activity	Thaumatin-like protein	Kontogiorgos et al. (2007)
*Bi*CHT-1	Smooth brome (*Bromus inermis*)	Hexagonal bipyramidal ice shaping	Chitinase	Nakamura et al. (2008)
*Rs*AFP	Japanese radish (*Raphanus sativus*)	TH = ∼0.2°C at 40 μg/mL, IRI activity, hexagonal ice shaping	None reported	Kawahara et al. (2009)
*PaAFP*	Norway spruce (*Picea abies*)	TH = 2.19°C at 400 μg/mL, bipyramidal ice crystals	Chitinase	Jarzabek et al. (2009)
*Pp*AFP	Blue spruce (*Picea pungens*)	TH = 2.02°C at 400 μg/mL, bipyramidal ice crystals	Chitinase	Jarzabek et al. (2009)
*Cp*CHT-AFP	Wintersweet (*Chimonanthus praecox*)	TH = 0.52°C at 1.5 mg/mL, hexagonal bipyramidal ice crystals	Class I endochitinase	Zhang et al. (2011)
*Ll*AFP1-3	Chinese privet (*Ligustrum lucidum*)	TH = 0.38-0.68°C at 5 mg/mL	None reported	Cai et al. (2011)
*Hr*CHT-1a/b	Seabuckthorn (*Hippophae rhamnoides*)	TH = 0.19°C at 0.2 mg/mL, high IRI activity, hexagonal ice shaping	Class I endochitinase	Gupta and Deswal (2012)
*Ba*AFP-1	Malting barley (*Hordeum vulgare* L.)	TH = 1.04°C at 18 mg/mL	Alpha-amylase inhibitor protein	Ding et al. (2015)
*Bd*IRI1-7	Purple false brome *(Brachypodium distachyon*)	TH = ∼0.15°C at 0.5 mg/mL, high IRI activity, hexagonal bipyramidal crystals	Phytosulfokine receptor kinase	Bredow et al. (2016b)

It is extraordinary that until recently there was no formal proof that IBPs confer freeze tolerance to their hosts, in that no gene knockdown had been made in any organism. The first knockdown of ice-binding activity from any organism was achieved in the purple brome grass *Brachypodium distachyon*, using a microRNA construct designed to attenuate several IBPs. These genetically engineered knockdown lines had 13–22% more membrane damage than wild-type plants following freezing to -10°C. Two of the knockdown lines also showed significantly reduced whole-plant freezing survival at -8°C, compared to the wildtype grass (Bredow et al., 2016b). These experiments have unequivocally confirmed the importance of IBPs in plant freezing survival.

### IBPs as Anti-pathogenesis Proteins

A large number of plant IBP genes show sequence homology to PR genes (**Table 1**). Of the six IBPs in winter rye, *Secale cereale*, two were identified as β-1,3-endoglucanases, two as class I and class II endochitinases, and two as thaumatin-like proteins, suggesting more than just a role in low temperature protection (Hon et al., 1995). These proteins have both ice-binding and hydrolytic activity (Yeh et al., 2000). This presents an interesting evolutionary context for the role of IBPs as dual function proteins, providing protection against both freezing and pathogen attack. Indeed, IBPs with homology to chitinase gene sequences have been identified in brome (*Bromus inermis)*, wintersweet (*Chimonanthus praecox*), and spruce (*Picea abies* and *P. peungens*) (Nakamura et al., 2008; Jarzabek et al., 2009; Zhang et al., 2011). A thaumatin-like protein has also been identified in winter wheat, *Triticum aestivum* (Kontogiorgos et al., 2007). Notably, aside from the IBPs from winter rye, the hydrolytic activity of these proteins has been largely untested.

Other proteins, including those from carrot, *Daucus carota* (Meyer et al., 1999), and sea buckthorn, *Hippophae rhamnoides* (Gupta and Deswal, 2012), share sequence homology with poly-galacturonase inhibitor proteins (PGIPs), although they may no longer possess PGIP activity, as has been shown for the carrot IBP, *Dc*AFP (Zhang et al., 2006). As well, freeze-tolerant grasses from the Pooideae subfamily share some sequence homology with leucine-rich repeat (LRR) phytosulfokine receptor tyrosine kinases (PS-RTKs), implicated in the recognition of pathogen-associated molecular patterns (Sandve et al., 2008). However, the carboxyl terminal kinase domains of these proteins have been replaced with ice-binding motifs and the amino terminal LRR domain lacks close conservation with the rice, *Oryza sativa*, PS-RTK presumed to be the gene of origin. Thus, it is unclear whether these proteins still serve a role in the perception of plant pathogens.

Ice-binding proteins serve an additional role in anti-pathogenses by inhibiting ice-nucleation activity (INA). Some bacteria appear to promote ice crystal growth as a method of dispersal (Wu et al., 2009), however, it is likely that epiphytes have employed this property in order to gain access to intracellular nutrient stores (Lindow et al., 1982). Ice-nucleation proteins (INPs) have been identified in Gram-negative bacteria including *Pseudomonas syringae, P. viridiflava, P. flourescences, P. borealis, Xanthomonas campestries, Erwinia ananas, Er. uredovra*, and *Er. herbicola* (Warren and Corotto, 1989; Hirano and Upper, 1995; Wu et al., 2009). Such ice-nucleation active (INA+) bacteria have been isolated from the leaves of numerous crop species and undoubtedly result in crop losses worth millions of dollars each year (Hill et al., 2014). Plant IBPs have been shown to lower the nucleation temperature and propagation rate of ice formed in the presence of *P. syringae* (Griffith et al., 2005). Moreover, an IBP from perennial ryegrass (*Lp*AFP), and the closely related purple brome grass, *B. distachyon* (*Bd*IRI), lowered the nucleation temperature of solutions containing *P. syringae* extracts by ∼1.9°C and ∼2.3°C, respectively (Tomalty and Walker, 2014; Bredow et al., 2017). Given the low TH activity of these proteins (∼0.3°C and ∼0.1°C, at the concentrations used), freezing point depression cannot explain the inhibition of bacterially derived INA. Although IBPs co-localized with INPs on the bacterial surface, these experiments are as yet only suggestive that IBPs interact directly with INPs, thereby preventing the growth of ice across INP surfaces.

## Ice-Binding Proteins: Mechanisms of Action and Observations

### Ice Crystal Adsorption and Shaping

**FIGURE 2:**
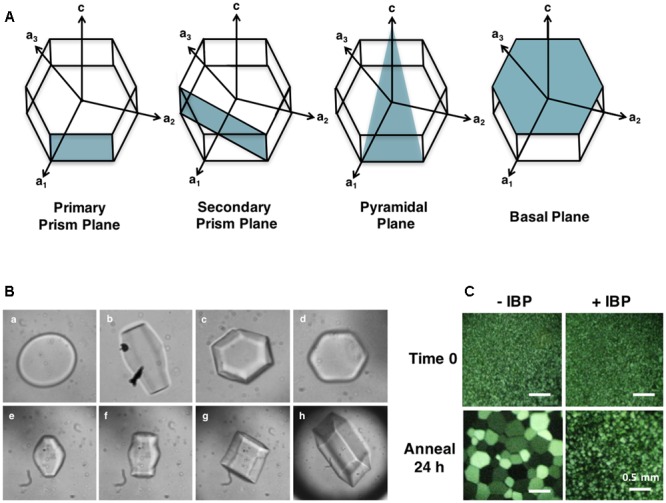
**(A)** Ice crystal planes adsorbed by IBPs. Plant IBPs have demonstrated affinity for primary and secondary prism planes, as well as basal planes. No plant IBPs have yet been identified with pyramidal plane affinity. *Lp*AFP, for example, binds both primary prism and basal planes. **(B)** Ice crystal morphologies in the absence **(a)** and presence of an IBP from two grasses (**b–h**). *Lolium perenne Lp*AFP (1 mg/mL) ice crystal shaping with the a-axes facing toward the reader is shown in **B**, and with the c-axis toward the reader in **c**. Shaping in the presence of low concentrations of IBP from *Brachypodium distachyon* (0.01 mg/mL) is shown in **d**. Panels (**e–h**) show an ice crystal burst as the equilibrium freezing point is exceeded. **(C)** Ice crystal growth following a 24 h annealing period at -6°C demonstrating ice recrystallization inhibition (IRI) by IBPs. Buffer without IBPs (–IBP) contains 100 mM Tris-HCl and 50 mM NaCl. Samples with *L. perenne* IBP (*Lp*AFP) (+IBP) were diluted to 0.5 mg/mL in the same buffer.

The activity of IBPs results from their ability to adsorb to ice, a process that can be visualized by monitoring the “shaping” of ice in the presence of IBPs. Water or standard buffers will freeze as circular disks that expand quickly from the prism plane at temperatures close to the freezing point (Nada and Furukawa, 2005). In contrast, adsorption of IBPs to one or more ice faces (**Figure 2A**) results in ice morphologies that are indicative of their ice plane specificity. These morphologies can be readily examined under a microscope (∼ 40 X) on a cooled stage (**Figure 2B**).

Moderately active IBPs shape ice into sharp bipyramidal crystals by adsorbing to prism planes (Bar-Dolev et al., 2012). Once the freezing point is reached, these crystals “burst” quickly from the c-axes forming needle-like structures. Hyperactive IBPs, on the other hand, are able to adsorb to more than one plane of ice, resulting in hexagonal bipyramidal or “lemon-shaped” ice crystals. In contrast to moderately active IBPs, ice crystals burst in a more gentle morphology, normal to the c-axis indicating basal plane affinity in addition to adsorption along the prism or pyramidal planes. This “protection” of the basal plane is hypothesized to be at least partially responsible for the higher activity of these proteins, since ice crystal growth is faster along the c-axis. Somewhat surprisingly, low TH plant IBPs, such as *Lp*AFP, direct ice crystal morphologies and burst patterns closer to that seen with hyperactive IBPs (**Figure 2B**). These ice crystals shape as hexagonal bipyramids that instead burst from the a-axes resulting in expansion of the basal planes, clearly indicating high affinity for this site. At low protein concentrations, low TH IBPs take on a mild hexagonal shape (**Figure 2B**), as seen in the cell extracts of cold-acclimated plants. This ice shaping is consistent for all plant IBPs identified to date with some minor differences in observed burst patterns, such as that seen with natively produced winter rye and purple brome IBPs, which burst from the tips of the prism plane, resulting in flower-shaped crystals (Yaish et al., 2006; Bredow et al., 2016b). Flower-shaped bursts have also been observed with some other plant extracts including natively produced *Lp*AFP.

### Thermal Hysteresis and Ice-Recrystallization Inhibition

In the absence of solutes and other modifiers, melting and freezing temperatures of solutions are at equilibrium. Unlike solutes that depress the freezing point below equilibrium in a colligative manner, IBPs can have a greater effect on the freezing point even at micromolar concentrations (Olijve et al., 2016). The adsorption-inhibition model has been used to describe this phenomenon such that the irreversible adsorption of IBPs to the surface of ice at specific intervals restricts the area where water molecules can join the ice crystal lattice to points between adjacent IBPs (Raymond and DeVries, 1977). This in turn, causes a curvature that is energetically unfavorable for ice crystal growth. With the ice crystal unable to grow further at this temperature, the liquid surrounding the ice remains in a supercooled state, resulting in a depression of the freezing point. If the temperature is lowered, the ice-water interface overcomes the energy barrier set in place by the minimum curvature of the ice crystal surface and growth ensues. The curvature on ice crystal surfaces cannot be visualized, however, the temperature at which a single ice crystal melts and grows in the presence of IBPs can be accurately determined using a nanoliter osmometer or equivalent device (Middleton et al., 2014).

As described by the Ostwald ripening effect, ice-recrystallization occurs because the growth of large ice crystals at the expense of smaller ones is a thermodynamically favorable reaction (Knight and Duman, 1986). This property can be assayed by snap freezing a solution at homogenous nucleation temperatures, so as to generate a field of tiny ice crystals, and subsequently annealing at temperatures close to the melting point (Middleton et al., 2014; **Figure 2C**). It is thought that similar to TH activity, IRI occurs by preventing the addition of water molecules from the quasi-liquid layer into the ice crystal lattice. However, IBPs likely also “pin” the surfaces of ice crystals, and as such, inhibit melting.

Thermal hysteresis and IRI are distinct properties with little correlation between measures of high IRI and high TH activity, as exemplified by plant AFPs which exhibit high IRI and low TH. However, the mechanisms that distinguish these ice-associating properties are currently unclear. It is worth noting that unlike TH, which requires a relatively high concentration of IBPs, IRI occurs at submicromolar concentrations (Yu et al., 2010), suggesting that freeze-tolerant plants may not require the high titres of IBPs required by freeze-susceptible organisms.

### Principals of Ice-Binding

The mechanism underlying IBP adsorption to ice has been debated for decades. Early evidence suggested that hydroxyl groups on the surface of IBPs could generate hydrogen bonds between the protein and the ice crystal lattice (Knight et al., 1993). It was later hypothesized that the hydrophobicity of IBP surfaces could facilitate adsorption through an entropy-driven reaction favoring ice binding over a solvent exposed surface (Sönnichsen et al., 1996). Currently, the anchored clathrate waters hypothesis is the most accepted model for ice crystal adsorption (Garnham et al., 2011). This model suggests that the IBP structure itself is responsible for the organization of surrounding water molecules into an ice-like lattice, by forming water cages around the methyl groups of outward facing residues on the ice-binding surface. This model is consistent with the ability of IBPs to also adsorb to gas hydrates (Sun et al., 2015; Walker et al., 2015). By forming structures that resemble the quasi-liquid layer existing between water and ice, IBPs are then able to merge with ice crystal surfaces. In this model, the protein will eventually become “frozen” to the surface after merging, resulting in irreversible adsorption.

It has been experimentally determined that IBPs adsorb to ice using a flat plane capable of forming water cages, known as an ice-binding “surface” or “face” (IBF). The properties of the IBF dictate how the water molecules will be organized and thus which plane of ice will be bound. This binding hypothesis has been considerably strengthened through protein crystallography which showed tightly bound water molecules on the proposed IBFs of crystallized bacterial and fish IBPs (*Mp*AFP and Maxi). These structures support the contention that IBFs organize surface waters with perfect complementarity to the primary prism plane (Garnham et al., 2011; Sun et al., 2014). Since many IBPs crystallize as dimers along their IBF, such analysis is not possible for many of these, including *Lp*AFP.

### Structural Models of Plant IBPs

As noted, a few dozen IBPs from plants have been characterized. Of the 15 that have been sequenced (**Table 1**) a crystal structure has only been obtained for *Lp*AFP, although partial molecular dynamic models have been proposed for *Dc*AFP and *Bd*IRI (Zhang et al., 2006; Bredow et al., 2017). Since IBPs appear to have evolved independently, there is no consensus sequence for IBPs, although some authors have strived to derive search algorithms (e.g., Yu and Lu, 2011). Generally, it has been established that IBFs are largely flat, often with repetitive residues bearing short, relatively hydrophobic R groups in comparison to non-IBFs, which frequently contain charged solvent-exposed residues (Davies and Hew, 1990; Garnham et al., 2011). The repetitive nature of many IBPs may facilitate the organization of waters into an ice-like lattice (Tyshenko et al., 1997; Garnham et al., 2008; Middleton et al., 2009; Zhuang et al., 2012). The IBFs identified in the few known IBPs with low TH, such as *Lp*AFP and *Bd*IRI, although also flat and repetitive, appear to have less regular IBFs with more substitutions of seemingly less ideal residues than that observed with hyperactive IBPs, possibly explaining their lower TH activity.

**FIGURE 3:**
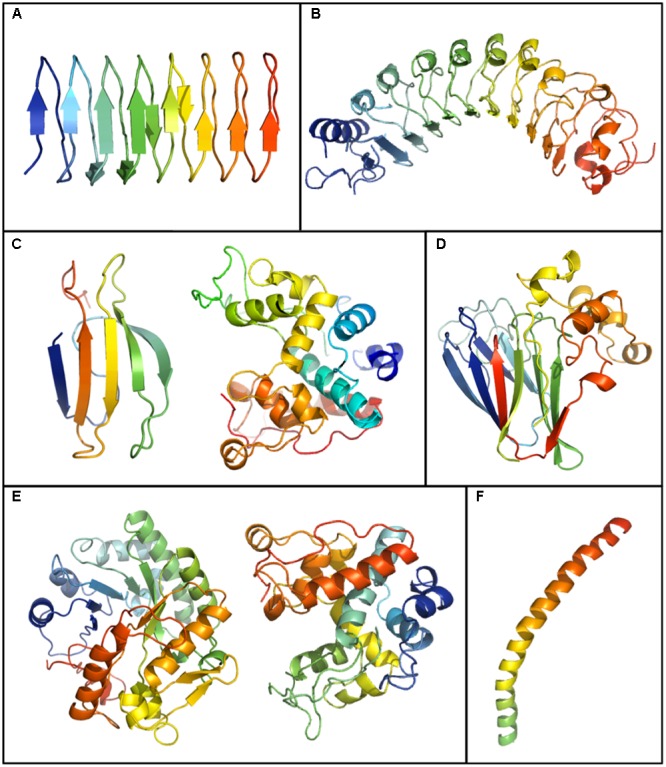
Plant IBP models generated by running GenBank sequences through the Phyre 2.0 server (http://www.sbg.bio.ic.ac.uk/phyre2/html/page.cgi?id=index). Shown is the crystal structure of the IBP domain of *Lolium perenne, Lp*AFP (GenBank ID: AJ277399.1) **(A)** and models for: *Daucus carota Dc*AFP (AF055480.1) **(B)**, a WRKY-IBP (STHP) (AAL268542.1) (left) and a type I endochitinase-IBP (Q84LQ7) (right) from *Solanum dulcamara* **(C)**, *Triticum aestivum* thaumatin like-IBP (AAM15877.1) **(D)**, a glucanase-IBP (CAJ58506.1) (left) and a type II endochitinase-IBP (right) (AF280438.1) from *Secale cereal*e **(E)**, and an IBP from *Chorella vulgaris* (ABR01234.1) **(F)**.

Although, as indicated, the crystal structures of plant IBPs are unknown, except for the truncated recombinant *Lp*AFP, modeling suggests that IBPs from disparate plants can form beta-sandwiches, alpha-helical folds, and globular structures (**Figure 3**). *Lp*AFP has a right-handed beta-helix with 7 loops consisting of 14–15 residues per turn with the repeated ice-binding motif NXVXG/NXVXXG, where X is a solvent exposed-residue (Middleton et al., 2012). Overall, *Lp*AFP is rather hydrophilic compared to other IBPs, with an abundance of outward facing residues that interact with ice including threonine, serine and valine (Middleton et al., 2009). The protein core is stabilized by two rows of hydrophobic asparagine residues. Structural modeling initially suggested two putative IBFs both with relatively flat surfaces (Kuiper et al., 2001), however, protein crystallography revealed that one of these faces was less planar. Site-directed mutagenesis confirmed that only the flatter “a-face” was involved in ice-binding (Middleton et al., 2009). In contrast, both of these faces appeared to bind ice in the closely related IBP *Bd*IRI, however, the flatter “a-face” appeared to be most necessary (Bredow et al., 2017).

Recombinant *Lp*AFP is a partial protein but the corresponding gene appears to encode both an amino-terminal LRR domain and a carboxyl-terminal ice-binding domain (Sidebottom et al., 2000). No crystal structure has been obtained for the full-length protein, but given that IBP activity is provided by the carboxyl ice-binding domain alone in apoplast extracts of *L. perenne*, it was hypothesized that hydrolysis occurred (Sandve et al., 2008). IBP nucleotide sequences from *B. distachyon* have similar bipartite structures as *Lp*AFP (Bredow et al., 2016b). Full-length recombinant LRR-IBP constructs were shown to have IBP activities. However, only the ice-binding domain of two IBPs, *Bd*IRI3 and *Bd*IRI4, were identified in the apoplast of cold-acclimated plants by mass spectrometry, further strengthening the hypothesis that IBPs are hydrolyzed *in planta.*

## Regulation of IBPs

### Cellular Localization

Given that ice-nucleation typically occurs outside cells, it is not unexpected that most IBPs have been isolated from apoplast extracts. Almost all plant IBPs are either predicted to be exported based on the identification of putative amino-terminal signal sequences, or have confirmed apoplastic localizations (**Figure 1B**; Griffith et al., 1992; Hon et al., 1994; Antikainen et al., 1996; Bredow et al., 2017). However, the dual function activities of some IBPs suggests a possible role for intracellular localization. For example, an IBP from the weeping forsythia (*Fs*AFP; *Forsythia suspensa*), that shares homology with dehydrins, is retained in the cytoplasm (Simpson et al., 2005). Additionally, *S. dulcamara*, produces an IBP that lacks an amino-terminal signal peptide and shares homology with a WRKY transcription factor that has been implicated in transcriptional regulation of PR genes (STHP-64; Huang and Duman, 2002). This suggests that certain plant IBPs might also act intracellularly to inhibit cellular dehydration or to regulate gene expression while still serving to prevent intracellular ice-nucleation.

Despite a few exceptions, most IBPs contain amino-terminal signal peptides that direct secretion to the apoplast through the Golgi–endoplasmic reticulum (ER) pathway (**Figure 1B**). An obvious exception is an IBP from *L. perenne, Lp*IRI2, which has no sequence upstream of the ice-binding domain (Sandve et al., 2008). Curiously, despite this lack of any recognizable signal peptide, transgenic studies in *A. thaliana* indicated that this isoform was nevertheless localized to the apoplast (Bredow et al., 2016a). Although the specific mechanism is unknown, nonconventional secretion pathways such as direct translocation channels, endosomal recycling, or membrane blebbing have been reported in plants (Cheng and Williamson, 2010). Non-Golgi secretion pathways are predicted to be responsible for ∼60% of the *A. thaliana* secretome, composed largely of stress-related proteins, which remain unidentified for the most part (Regente et al., 2012). It is possible that some non-classical secretion pathways could allow more rapid secretion of proteins and could provide a selective advantage in stressful environments (Keller et al., 2008; Bredow et al., 2016a).

It has been argued that intracellular localization of IBPs could be advantageous. However, the *in planta* expression of a partial IBP (*Lp*AFP) lacking a signal peptide, and retained in the cytoplasm of transgenic plants, provided significantly less freeze protection compared to apoplastically localized *Lp*IRI2 and *Lp*IRI3 (Bredow et al., 2016a). This disparity cannot be explained by differing ice-associating activities since *in vitro* assays with the respective recombinant proteins were comparable. Thus, the observation of striking differences in transgenic protection depending on the IBP expressed argues that plants are only afforded significant frost protection when localized to the apoplast. Presumably protection is provided by preventing the growth of large, damaging ice crystals that could rupture plasma membranes. Although rare, intracellular ice could result in irreparable cellular damage (Steponkus, 1984; Guy, 1990; Pearce and Ashworth, 1992; Wisniewski et al., 1997). Thus, the production of any endogenous intracellular IBPs would likely be disadvantageous. Although it is possible that intracellular plant IBPs could theoretically provide protection through membrane stabilization or opportunistically through the release of IBPs following cell lysis and the subsequent protection of neighboring cells, such mechanisms have not been experimentally demonstrated.

### Spatial–Temporal Regulation

Plant IBPs are not constitutively expressed but rather are induced in response to various stimuli. Cold acclimation is an invariable regulator of IBP expression in plants, however, there is variability in the time required at a low temperature for maximal IBP expression. IBP transcripts from *L. perenne* for instance, accumulate in as little as 1 h of exposure with maximal expression after 7 days (Zhang et al., 2009). Similarly, *Dc*AFP transcripts accumulate in as little as 30 min at 4°C (Meyer et al., 1999). IBPs from the same plant may also be differentially regulated as indicated by the observation that winter rye glucanase-IBP transcripts are abundant after 24 h at 5°C while chitinase-IBPs from the same plant require 3–7 weeks for full induction (Hon et al., 1995; Yeh et al., 2000). The nature of the cold treatment also impacts IBP induction as demonstrated by the induction of winter wheat (*T. aestivum*) IBPs *Ta*IRI1 and *Ta*IRI2 by both cold-shock and gradual temperature decreases, whereas chitinase-, glucanase-, and thaumatin-like IBP transcripts were only induced by the latter regime (Winfield et al., 2010).

In the field, cold acclimation involves both low temperatures and short photoperiods. This has been observed with winter rye IBPs, which accumulate more rapidly under short day cycles (8 h light) that more closely resemble autumn conditions, than after long days (16 h light) (Marentes et al., 1993). It is possible that IBP turnover may be facilitated by temperature changes as seen with cold-induced chitinase-IBPs which degrade 12 h after exposure to 20°C, or *Bd*IRIs which are inactivated at temperatures above 4°C (Hon et al., 1995; Bredow et al., 2017). *Lp*AFP denatures at temperatures >25°C but can refold when returned to low temperatures, at least *in vitro* (Sidebottom et al., 2000). Although these observations suggest changes in protein titers, many plant IBPs may not be subjected to rapid circadian cycle turnover as suggested by IBPs from winter wheat, sea buckthorn and the Tibetan herb, *Rhodiola algida*, all of which are relatively thermostable (Lu et al., 2000; Gupta and Deswal, 2012; Kontogiorgos et al., 2007).

Some IBPs are induced by other abiotic stress, hormones, or biotic stimuli. For example, in the absence of cold acclimation, winter rye IBPs accumulate after treatment with an ethylene-producing compound, ethephon (Yu et al., 2001). These same IBPs can be induced with a variety of stress treatments including salicylic acid (SA), abscisic acid (ABA), pathogen attack, and drought (Yu and Griffith, 2001; Yu et al., 2001). While “IBPs” induced by SA or ABA lack ice-binding activity and thus their designation is uncertain, both drought and ethylene treatments are associated with the presence of IBPs that efficiently adsorbed to ice. As well, IBPs secreted in *R. algida* suspension cells following ABA induction also showed ice-binding activity (Lu et al., 2000). This research provides an interesting context for dual-function proteins, suggesting that the nature of the inducing stimuli can influence their ice-associating activity. The presence of enzyme substrates or cellular targeting could allow involvement in differential functions. However, it has also been hypothesized that multiple roles of the same IBP could be explained by alternative protein folding or post-translational modifications (Yaish et al., 2006; Gupta and Deswal, 2014b).

The developmental stage of plants can also be important for IBP induction (Griffith and Yaish, 2004). IBPs in leaf tissues of cold-acclimated winter rye show higher levels when the plants developed under conditions of cold acclimation, in contrast to plants that were fully developed prior to exposure to low temperatures (Hon et al., 1995; Yeh et al., 2000). Additionally, not all IBPs are expressed concordantly; in winter rye, glucanase IBPs are induced before thaumatin-like IBPs, while chitinase-IBPs are expressed much later. While this profile could indicate that some IBPs require different low temperature exposure periods, since chitinase-IBPs only accumulate in tissues which develop at acclimation temperatures and not in plants subsequently transferred to cold, a developmental component is more likely (Yeh et al., 2000). Although seeds have been reported to avoid freezing using polyols and sugars, an annual cultivar of purple brome contains ice-binding activity in the seeds, presumably *Bd*IRIs (Bredow, 2017). More curious perhaps is the presence of cold acclimation-dependent, ice-binding activities in senescing tissues such as the leaves on the annual shoots of the goldenrod, *Solidago canadensis*, which overwinters as a rhizome (Tomalty, 2016). Clearly more research is warranted to determine the presence and possible functions of IBPs at various developmental stages.

Ice-binding proteins from different plant hosts also show distinctive tissue-specific expression patterns. For instance, chitinase-IBPs from winter rye localized to the parenchymal sheath, mesophyll, epidermal and phloem cells of leaves, while no activity was detected in roots (Pihakaski-Maunsbach et al., 1996). This distinct localization is in contrast with *Dc*AFP, which accumulated equally in leaves, stems and roots following cold acclimation (Smallwood et al., 1999). Even different isoforms can be independently regulated as demonstrated by the ubiquitous winter wheat *Ta*IRI-1, compared to the leaf-specific expression of *Ta*IRI-2 from the same plant (Tremblay et al., 2005). Differential isoform expression is also likely in *B. distachyon* with two of 7 isoforms identified in 2 day cold-acclimated leaves (Bredow et al., 2016b).

### Post-translation Modification

As their name implies, most ice-binding activity identified in plants is associated with proteins, several of which are thought to be glycosylated (**Figure 1B**). Glycosylated IBPs have been identified in disparate plants including *L. perenne, S. dulcamara, D. carota*, the Mongolian Menkhargana (*Ammopiptanthus mongolicus*), and *H. rhamnoides* (Duman, 1994; Smallwood et al., 1999; Sidebottom et al., 2000; Fei et al., 2008; Gupta and Deswal, 2012). In some cases, glycosylation is a requirement for ice-binding activity, such as for IBPs produced in *S. dulcamara* (Duman, 1994), while other plants do not require such modifications. For example, *Lp*AFP, produced recombinantly in *E. coli*, and thus having no post-translational modifications, still retains IRI, TH and ice-shaping capabilities (Pudney et al., 2003). The same is true for *D. carota* and *H. rhamnoides* (Smallwood et al., 1999; Gupta and Deswal, 2014a).

Initially, it was hypothesized that post-translational modification could play a role in the regulation of dual function IBPs, specifically those with ice-binding and hydrolytic activities (Yaish et al., 2006). Hydroxylation, for example, was reported in chitinase-IBPs of winter rye but this appears to have no effect on ice-binding (Sticher et al., 1992; Hon et al., 1995; Yeh et al., 2000). More recent data has implicated conformational changes in different IBP activities, as seen with the chitinase-IBPs from *H. rhamnoides*, that appear to exhibit Ca^2+^-dependent refolding (Gupta and Deswal, 2014a). However, class-I chitinase IBPs from brome, *B. inermis*, are not affected by Ca^2+^ (Nakamura et al., 2008) and winter rye IBPs are inhibited by high Ca^2+^ concentrations (Stressmann et al., 2004). Similar to many other regulatory factors implicated in IBP activity, the role of Ca^2+^ is still not clear, although this ion does play a role in the appropriate folding of a brine-lake bacterial IBP (*Mp*AFP; Guo et al., 2012).

## Prospects for the Use of IBPs for Transgenic Crops

Considerable efforts have been employed to generate plants with enhanced freeze tolerance, mostly by traditional breeding methods but also through the investigation of transgenics. The latter approach has been met with notably limited success. These efforts are important since changes in atmospheric circulation and climate change influence the timing of frost-free periods in the spring and autumn, resulting in unpredictability (Strong and McCabe, 2017). Climate change in particular appears to be responsible for changes in the timing of autumn frosts making growers uncertain with respect to crop management. Theoretically, expression of IBPs should provide freeze protection to sensitive crops exposed to seasonally sub-zero temperatures. Such approaches would be most applicable to plants without intrinsic abilities to cold acclimate, for example certain annual garden plants or tender ornamentals for the cut flower industry, as well as crops with a limited potential for frost tolerance such as some soft fruits. The controlled expression of transgenic IBPs could reduce freeze-induced cellular dehydration and membrane damage. Alternatively, in situations where temperatures rapidly drop below zero, without the necessary cold acclimation periods, as can occur in prairie climates, constitutive expression of IBPs may limit near-lethal tissue damage, and might be attractive to the floral industry, which may be less concerned with genetic modifications.

Attempts have been made to use IBPs found in disparate host organisms in order to provide freeze protection (**Table 2**). Initial efforts focused on the expression of fish IBPs with moderate levels of TH activity in tobacco *(Nicotiana tobacum*) and tomato (*S. lycopersicum*) (Hightower et al., 1991; Kenward et al., 1999). Although TH and IRI were observed in tissue extracts, when plants were tested for their freezing tolerance capabilities, they showed little frost resistance and only lowered the lethal temperature by 1°C (Wallis et al., 1997; Worrall et al., 1998; Kenward et al., 1999; Khanna and Daggard, 2006). The use of hyperactive IBPs from spruce budworm and fire-colored beetle in tobacco and *A. thaliana* were expected to lower the freezing point significantly and thus avoid freezing. Although these experiments showed that IBPs accumulated and exhibited both TH and IRI activity, enhanced freeze survival was not observed (Holmberg et al., 2001; Huang et al., 2002). Likely due to their low TH activities, attempts to express transgenic plant IBPs in freeze-susceptible host plants were only later reported. Remarkably, even today only three studies have used plant IBPs, derived from carrot and perennial ryegrass, to develop transgenic plants. In all cases the transgenic plants demonstrated decreased electrolyte leakage, indicating membrane protection, and enhanced freeze survival at temperatures below –5°C, depending on the sequence and the host (Fan et al., 2002; Zhang et al., 2010; Bredow et al., 2016a).

**Table 2:** Transgenic plants expressing ice-binding proteins and antifreeze proteins (AFPs).

Protein	Protein origin	Host plant	Transgenic phenotype	Reference
afa-3	Winter flounder (*Pseudopleuronectes americanus*)	Tobacco (*Nicotiana tobacum*)	IBP accumulated in extracts with truncated protein (Spa-Afa5)	Hightower et al. (1991)
		Tomato (*Solanum lycopersicum*)		
Type I afp	Winter flounder (*Pseudopleuronectes americanus*)	Tobacco (*Nicotiana tobacum*)	Protein accumulated at 4°C	Kenward et al. (1999)
Type I afp	Winter flounder (*Pseudopleuronectes americanus*)	Potato (*Solanum tuberosum*)	Reduced electrolyte leakage; lowered LT_50_ by 1°C	Wallis et al. (1997)
*Dc*AFP	Carrot (*Daucus carota*)	Tobacco (*Nicotiana tobacum*)	Accumulated antifreeze activity in apoplast	Worrall et al. (1998)
Type II afp	Sea raven (*Hemitripterus americanus*)	Tobacco (*Nicotiana tobacum*)	Protein accumulated; no frost resistance	Kenward et al. (1999)
CfAFP	Spruce budworm (*Choristoneura fumiferana*)	Tobacco (*Nicotiana tobacum*)	IBP accumulated in apoplast; extracts exhibited TH/IRI	Holmberg et al. (2001)
dAFP-1	Fire-colored Beetle (*Dendroides canadensis)*	*Arabidopsis thaliana*	AFP accumulated in apoplast; lowered freezing temperature but did not enhance freeze survival	Huang et al. (2002)
*Dc*AFP	Carrot (*Daucus carota*)	Tobacco (*Nicotiana tobacum*)	Exhibited chilling tolerance at -2°C	Fan et al. (2002)
*Mp*AFP149	Desert beetle (*Microdera punctipennis*)	Tobacco (*Nicotiana tobacum*)	Accumulated in apoplast; enhanced cold tolerance at -1°C	Wang et al. (2009)
TaAFPI	Winter flounder *(Pseudopleuronectes americanus*)	Winter wheat (*Triticum aestivum*)	Protein accumulated in apoplast; reduced electrolyte leakage	Khanna and Daggard (2006)
THPI	Spruce budworm (*Choristoneura fumiferana*)	*Arabidopsis thaliana*	Reduced electrolyte leakage	Zhu et al. (2010)
*Lp*IRIa *Lp*IRIb	Perennial ryegrass cv. Caddyshack (*Lolium perenne*)	*Arabidopsis thaliana*	Reduced electrolyte leakage at temperatures below -8°C; enhanced survival at temperatures between 4 and -8°C	Zhang et al. (2010)
*Lp*AFP *Lp*IRI2 *Lp*IRI3	Perennial ryegrass v. Pacific Seed Diploid (*Lolium perenne*)	*Arabidopsis thaliana*	Reduced electrolyte leakage by 12–39% at -6°C; enhanced survival at temperatures between -5 and -8°C.	Bredow et al. (2016a)

These successful experiments suggest that IBPs that have evolved from freeze-tolerant organisms could be better suited for transfer to other plants and the generation of transgenic crops. This may be true for a number of reasons. As mentioned earlier, ice formed in the presence of moderately active IBPs can produce sharp bipyrimidal ice crystals that result in spicular ice shards as they burst, presumably causing significant cellular damage. It is also likely that if freezing points are lowered significantly with the use of a high TH AFPs, explosive ice crystal growth could risk dangerous intracellular freezing. Interestingly, an IBP from the Antarctic microalga, *Chloromonas sp.*, has recently been identified that has remarkably similar properties to that seen with plant IBPs, with a low TH (∼ 0.4°C at 5 mg/mL) and hexagonal ice-shaping (Jung et al., 2016). We suggest that IBPs from algal species, such as that described above, may also be good candidates for transgenics. An added advantage of plant IBPs for crop improvement is their ability to suppress ice-nucleation associated with pathogenic epiphytes, a property that has not been observed for non-plant IBPs (Tomalty and Walker, 2014; Bredow et al., 2017).

Recently, it has been demonstrated that expression of more than one IBP isoform, as would be expressed endogenously in plants, further enhanced freeze survival in transgenic *A. thaliana* (Bredow et al., 2016a). Thus, IBPs may work cooperatively to optimally restrict ice crystal growth, by preferentially adsorbing to different ice crystal planes or through some other unknown mechanism. In conjunction with the observation that protein localization dictates the level of freeze-protection afforded to plants, it is clear that further research regarding the regulation of IBPs in plants is needed before such transgenic produce could be of practical agricultural value. The lack of a freeze-tolerant model organism for the study of IBP function has been one of the greatest limitations of this research. However, the recent identification and characterization of a family of plant IBPs that are required for protection against freezing damage (Bredow et al., 2016b), indicates that the model grass, *B. distachyon*, and other perennial species of *Brachypodium* will be a valuable tool for the study of plant IBPs in the future. The prospect that these extraordinary proteins could not only contribute to future food security, but also the ornamental flower industry, will make these efforts well worthwhile.

## Author Contributions

MB wrote this manuscript with revisions and editorial advice from VW.

## Conflict of Interest Statement

The authors declare that the research was conducted in the absence of any commercial or financial relationships that could be construed as a potential conflict of interest.
